# Robust Estimation of Unsteady Beat-to-Beat Systolic Blood Pressure Trends Using Photoplethysmography Contextual Cycles

**DOI:** 10.3390/s25123625

**Published:** 2025-06-09

**Authors:** Xinyi Huang, Xianbin Zhang, Richard Millham, Lin Xu, Wanqing Wu

**Affiliations:** 1 School of Biomedical Engineering, Sun Yat-sen University, Shenzhen 518107, China; huangxy533@mail2.sysu.edu.cn (X.H.); zhangxb55@mail2.sysu.edu.cn (X.Z.); 2 Department of Information Technology, Durban University of Technology, Durban 4001, South Africa; richardm1@dut.ac.za; 3 General Hospital of the Southern Theatre Command, Guangzhou 510010, China; 4 The First School of Clinical Medicine, Southern Medical University, Guangzhou 510515, China

**Keywords:** health monitoring, cardiovascular disease, blood pressure estimation, photoplethysmography, deep learning

## Abstract

Hypertension and blood pressure variability (BPV) are major risk factors for cardiovascular disease (CVD). Single-channel photoplethysmography (PPG) has emerged as a promising daily blood pressure (BP) monitoring tool. However, estimating BP trends presents challenges due to complex temporal dependencies and continuous fluctuations. Traditional methods often address BP prediction as isolated tasks and focus solely on temporal dependencies within a limited time window, which may fall short of capturing the intricate BP fluctuation patterns implied in varying time spans, particularly amidst constant BP variations. To address this, we propose a novel deep learning model featuring a two-stage architecture and a new input structure called contextual cycles. This model estimates beat-to-beat systolic blood pressure (SBP) trends as a sequence prediction task, transforming the output from a single SBP value into a sequence. In the first stage, parallel ResU Blocks are utilized to extract fine-grained features from each cycle. The generated feature vectors are then processed by Transformer layers with relative position encoding (RPE) to capture inter-cycle interactions and temporal dependencies in the second stage. Our proposed model demonstrates robust performance in beat-to-beat SBP trend estimation, achieving a mean absolute error (MAE) of 3.186 mmHg, a Pearson correlation coefficient applied to sequences ($R_{\mathrm{seq}}$) of 0.743, and a variability error (VE) of 1.199 mmHg. It excels in steady and abrupt substantial fluctuation states, outperforming baseline models. The results reveal that our method meets the requirements of the AAMI standard and achieves grade A according to the BHS standard. Overall, our proposed method shows significant potential for reliable daily health monitoring.

## 1. Introduction

Cardiovascular disease (CVD) remains the leading cause of mortality and illness worldwide [1]. Hypertension and blood pressure variability (BPV) are recognized as independent and modifiable risk factors for CVD and strong predictors of adverse events [2,3]. Independently of average BP, the magnitude and pattern of BP fluctuations define BPV [4]. Although variability naturally exists in BP, frequent excessive fluctuations, particularly elevated beat-to-beat systolic blood pressure (SBPV), may indicate underlying premature vascular lesions [5,6]. Moreover, the abrupt surges in BP mostly occur in hypertensive patients with hypertensive emergency [7] or obstructive sleep apnea (OSA) [8], leading to target organ damage and increased risk of cardiovascular events, which are associated with significant morbidity and mortality [9,10,11]. Beat-to-beat BP trends can serve for BP values and BPV assessments on different temporal scales, especially for the detection of abnormal abrupt BP changes, which is critical for early prevention and timely intervention. Due to the limitations of intermittent measurements, cuff-based methods may fail to capture continuous BP fluctuations and detect potential abnormalities. Recently, there has been an increased focus on active health, leading to efforts to develop continuous BP monitoring using portable sensors.

Considering the burden of multiple sensors for portability, studies have attempted to achieve continuous BP monitoring based on the easily available single-channel photoplethysmography (PPG) only. PPG is a non-invasive, portable, unobtrusive, and cost-effective optical technique for measuring blood volume changes during the cardiac cycle [12] and is widely used in wearable devices. Physiological and pathological changes in the cardiovascular system can affect BP, leading to variations in PPG amplitude, frequency, and morphology. Therefore, PPG provides valuable information on cardiovascular status [13] and is commonly used for continuous non-invasive BP monitoring. However, the complex temporal dependencies and dynamics in BP make continuous BP monitoring from PPG signals challenging. First, BP fluctuation patterns and PPG waveforms evolve over time due to complex interactions among various factors and cardiovascular regulatory mechanisms [14,15]. Consequently, BP, PPG waveforms, and their relationship exhibit complex short-term and long-term temporal dependencies related to BP dynamics across time steps (within and between cardiac cycles) [16]. Secondly, the constantly fluctuating and non-stationary BP leads to a wide range of BP value distributions, with some trends being stable while others may surge [17]. Compared to the complex dynamic fluctuations in BP, changes in PPG waveform morphology are relatively subtle, posing challenges for continuous BP monitoring.

Existing studies have extracted numerous hand-crafted features to capture subtle variations in physiological signals related to cardiovascular conditions and blood flow characteristics. Khalid et al. [18] explored BP estimation using three significant features extracted from good quality PPG signals. They trained and compared three machine learning algorithms (regression tree, multiple linear regression, and support vector machine). This study further analyzed the performance under different BP categories, observing that better performance in the normotensive category. Fleischhauer et al. [19] estimated SBP values by extracting multiple features from the PPG and its derivatives and applying XGBoost regression. Through interpretation with Shapley additive values, they identified specific features crucial for BP estimation. The method was primarily tested on SBP and suggested to be applicable to diastolic BP (DBP). Gupta et al. [20] stated that the natural morphology of PPG indicates different BP circulation events, which challenges handling time-varying features and temporal dependencies. Thus, they considered using higher-order derivatives to extract the nonlinear features and using machine learning algorithms to map from these features to BP values. Liu et al. [21] extracted as many as 222 features from electrocardiogram (ECG) and PPG and attempted to identify features with a causal relationship rather than correlated with BP changes through the fast causal inference (FCI) algorithm. They integrated the causal inference mechanism with the BP model via the time-lagged link and developed a causal regression model based on causal features. This demonstrates that the complete cardiac cycle of physiological signals contains rich hemodynamics information effective for BP prediction. However, hand-crafted features may yield unreliable values and incomplete representations of BP dynamics. Furthermore, classical machine learning methods may struggle to capture temporal dependencies, particularly when BP fluctuates significantly [22]. In contrast, deep learning algorithms can automatically extract complex features from raw signals and efficiently capture temporal dependencies. Yu et al. [23] utilized the first and second derivatives of PPG signals as additional inputs to enrich the information related to the correlation between the waveform and BP. Attention-based residual improved U-Net was employed to determine the effective and non-redundant features. Huang et al. [24] proposed a new end-to-end framework based on MLP-Mixer and generated multi-channel inputs by integrating multiple filtering methods to fuse information. MLPlstm-BP with the LSTM embedding layer aims to extract the temporal features and shows better performance. Miao et al. [25] conducted experiments on patients with arrhythmia. They crop and pad each ECG signal segment into a fixed length of 300 points (containing three cardiac cycles) and output the average BP values of the three heartbeats. To capture both morphological and rhythmic features that could impact BP variation, ResNet and bidirectional LSTM are combined due to their superior performance in capturing time-related features. Wang et al. [26] utilized GRU to consider cardiovascular activity changes in feature vectors for temporal dependency, and an attention module was further used to filter BP-related features. They fused the demographic information into the attentive pooling module to improve the performance of SBP prediction. Liu et al. [27] proposed a BiGRU model with attention mechanisms to capture temporal dependences of the 10 s PPG for longer-term BP trends. Ma et al. [28] proposed an end-to-end SE-MSResUNet network that leverages the strengths of 1D-UNet, residual connections, and SE modules to extract important BP-related information and establish a correct mapping relationship between PPG and arterial blood pressure (ABP) signals.

Although these methods have had some success in predicting BP values, they may not be suitable for estimating beat-to-beat BP trends. First, existing methods map the extracted features within one cardiac cycle or overall time window to a single SBP value, treating each SBP prediction as an isolated task [18,19,20,21,22,23,24,25,26,27]. However, temporal dynamic dependencies can evolve due to changes in the environment, activities, and health status over time. Conventional single-step predictions cannot provide sufficient contextual multi-step information to explore underlying temporal patterns and transition trends in adjacent samples [29,30,31]. This approach may be insufficient for capturing fine-grained BP fluctuation patterns across different time spans. Secondly, most studies derive predicted BP values from the average of several consecutive beats within a specified period [23,24,25,26,27], assuming BP is nearly stable in the short term. However, individuals may have apparently normal or comparable average BP levels but still experience abnormal BP fluctuations [32]. Indeed, beat-to-beat BP provides detailed information on continuous BP variability and cardiovascular conditions [33]. Beat-averaged BP may overlook BP dynamics and even smooth out BP surges, which is not conducive to tracking BP fluctuations and detecting potential abnormalities.

Due to the elevated variability and greater changes in SBP dynamics, SBP estimation in heterogeneous populations has been proven to be a more complex and clinically significant challenge [34,35,36]. In this paper, beat-to-beat SBP trend estimation can be generalized as a sequence construction task, where the output label is turned from a single SBP value into a sequence. Patch-based input has been proven to be effective and superior in long-term time series prediction [37,38,39]. Decomposing complex time series into a series of consecutive temporal patches helps to enhance the representation of local semantics and provides context information in chronological order, which can improve the understanding and expression of complex problems and reveal the intricate temporal patterns in time series. Inspired by this, we introduce an input structure called contextual cycles, where PPG signals are divided into small segments according to cardiac cycles and arranged chronologically in channels. Reliable temporal dependencies modeling relies on fine-grained feature extraction related to cardiovascular activity within each PPG cycle, as well as on the interactions within and between contextual cycles to capture SBP fluctuation patterns. In this work, we propose an architecture for estimating beat-to-beat SBP trends, which consists of two stages based on contextual cycles. Specifically, in the intra-cycle feature extraction module (IFEM), parallel Residual U-like (RseU) Blocks are used to extract representative features from each PPG cycle. The feature vectors obtained from the IFEM are then fed into the context-sensitive interaction module (CIM), utilizing a Transformer and relative positional encoding (RPE) to explore inter-cycle temporal dependencies. The main contributions of our work are as follows:

- We develop a new approach for SBP trend estimation to transform the task into sequence prediction. Our method prioritizes beat-to-beat SBP variability for assisting in health applications, particularly for heterogeneous populations with frequent and dramatic fluctuations in SBP.
- We present a hybrid architecture based on the contextual cycles. ResU blocks extract hemodynamic information and enhance semantic representation. The patch-based structure provides temporal order and context for intra-cycle feature vectors, enabling a Transformer with RPE (Trans-RPE) that considers the relative temporal distance to explore inter-cycle interaction and more reliable temporal dependencies for complex SBP fluctuation patterns.
- We conduct studies with adequate variations in SBP, and the results demonstrate that our model excels in estimating the trend of beat-to-beat SBP with MAE and VE of 3.186 and 1.199 mmHg, especially in unsteady states. The classification accuracy for abnormal variations reaches 80.36%. Furthermore, our model meets the AAMI and BHS grade A standards.

## 2. Materials and Methods

### 2.1. Database and Data Pre-Processing

The proposed model in this study was developed based on the cuff-less blood pressure estimation dataset from the University of California, Irvine (UCI), Machine Learning Repository, which is a subset of the MIMIC-II [40]. The dataset contains 12,000 recordings of intensive care unit (ICU) patients, consisting of simultaneous, continuously recorded PPG and ABP signals at a sampling rate of 125 Hz. ABP is widely regarded as the gold standard for arterial pressure measurementand and carries valuable physiological information embedded in its waveform. SBP and DBP correspond to the peak and valley of the ABP waveform, respectively, offering clinically meaningful metrics for cardiovascular assessment.

In the data pre-processing stage, records of PPG and ABP signals with insufficient length (less than 8 min) were removed to ensure that the waveforms were long enough to contain dynamic fluctuations. To ensure a certain signal quality, anomaly detection was performed on both PPG and ABP signals, and low-quality segments were discarded. Studies have shown that Chebyshev II filters are more effective than other types of filters in improving the quality of PPG signals [41], so we employed a 4th-order Chebyshev II filter to process PPG signals.

Subsequently, the onsets of the PPG signals were identified using the peaks and valleys detector proposed by Zong [42]. PPG signals were segmented into non-overlapping slices by cardiac cycle. The length of every PPG cycle slice was adjusted to 125 by zero padding (when the length is less than 125 samples) or downsampling samples (when the length is greater than 125 samples). The SBP value for each cycle slice can be obtained from the peak of the corresponding ABP cycle.

We construct our dataset as *D* = {*C*, *S*}, where *C* represents contextual cycles and *S* represents SBP sequences. Each input $C_{i}$ (i.e., contextual cycles) consists of consecutive *n* cycle slices in chronological order from the same person, denoted as $C_{i}=\left[c_{i,1},c_{i,2},\ldots ,c_{i,n}\right]$, and the corresponding output is an SBP sequence $S_{i}=\left[{\mathrm{SBP}}_{i,1},{\mathrm{SBP}}_{i,2},\ldots ,{\mathrm{SBP}}_{i,n}\right]$, composed of *n* SBP values for *n* cardiac cycles. In this work, we set *n* to 60, since the BP surge is defined as BP fluctuations over tens of seconds. In particular, it is noted that the contextual cycles do not share samples from the same cardiac cycle.

**Figure 1:**
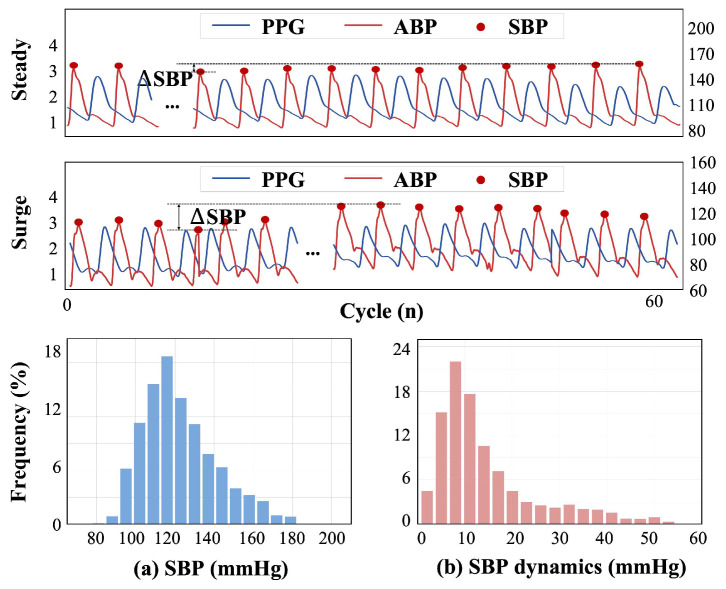
Distribution histograms of SBP and SBP dynamics: (**a**) the distribution of all the SBP values in the dataset; (**b**) the statistics of SBP dynamics, derived from the difference between the maximum and minimum values (*ΔSBP*) for each sequence.

Moreover, we set the criteria that each individual must have at least one sequence with the magnitude of SBP variations (*ΔSBP*) over 10 mmHg. The final processed dataset contains 3442 samples totaling 206,520 single beats from 238 subjects. For each subject, the samples were arranged in chronological order and non-overlappingly divided into three parts in a ratio of 7:1:2 for the training, validation, and test sets. Crucially, the training data always precedes the test data in time for each subject, ensuring strict temporal separation. Research [36] suggests that this setup maintains causality in the data and prevents data leakage. In particular, our dataset includes rich and sufficient BP fluctuations and appropriate changes in BP distribution, which enhance the reliability of the results using the personalized sample-based splitting method. The statistical information after these processes and segmentation is shown in Figure 1.

### 2.2. Network Architecture

**Figure 2:**
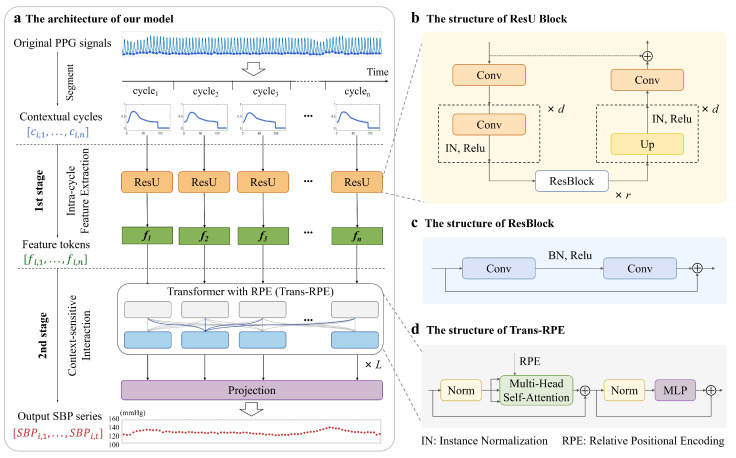
(**a**) The architecture of our proposed model with two stages: Intra-cycle Feature Extraction and Context-sensitive Interaction. (**b**) ResU Block. (**c**) ResBlock. (**d**) Transformer layer with RPE. For a given input, each PPG cycle slice of contextual cycles is first fed into ResU branches in parallel to generate a feature vector. Total *n* feature vectors are stacked into feature tokens in chronological order. Then, the contextual information between tokens interacts through Trans-RPE layers. Finally, the model outputs a SBP sequence.

Our model is proposed for beat-to-beat SBP trend estimation based on the contextual cycles. Specifically, given the dataset $D=\{\left(C_{1},S_{1}\right),\left(C_{2},S_{2}\right),\ldots ,\left(C_{i},S_{i}\right)\}$, we construct the SBP sequence $S_{i}$ from the contextual cycles $C_{i}$, where $C_{i}\in R^{n\times l_{0}}$ is the i-th input composed of *n* PPG cycles with the length of $l_{0}$ samples, and $S_{i}\in R^{1\times n}$ represents the i-th output containing *n* SBP values. For complex fluctuations in SBP, our approach relies on fine-grained extraction of cardiovascular-related features within each PPG cycle and on temporal dependencies modeling through contextual interactions between features obtained from different cycles. To this end, we propose a two-stage architecture, including IFEM and CIM. Figure 2 displays the specific model framework diagram. The training process has been presented in Algorithm 1.

#### 2.2.1. Intra-Cycle Feature Extraction

The IFEM aims to extract representative features independently from contextual cycles for rich hemodynamic information. For each PPG cycle slice in contextual cycles, we construct the U-like structure by stacking multiple convolutions and upsampling operations, which can bring multi-scale receptive fields as the deepening of convolutional layers to obtain the fine-grained blood flow information. Further, we introduce residual structures for complex feature fusion and representation. Meanwhile, residual structures excel in addressing the vanishing gradient problem, allowing for deeper network architectures and enhancing the stability of models. Thus, the ResU Block is a hybrid architecture that combines the U-like convolutional structure with residual blocks for extracting subtle changes in PPG signals within each cycle.

We utilize the parallel ResU Block branches to explore intra-cycle information from contextual cycles. Specifically, contextual cycles are $C_{i}\in R^{n\times l_{0}}$, where *n* denotes the total number of PPG cycles and equals 60, and $l_{0}$ is the size of each cycle slice and equals 128. Each cycle slice in contextual cycles $c_{i,j}\in R^{1\times l_{0}}$, j=1,…,n, is independently fed into ResU Block. This process is formulated as follows:

(1) $$f_{i,j}=f_{cir}\left(f_{up}^{d}\left(f_{res}^{r}\left(f_{down}^{d}\left(f_{cir}\left(c_{i,j}\right)\right)\right)\right)\right)+c_{i,j}$$

where $f_{cir}$ represents the convolution, instance norm, and ReLU function. *d* is the depth of U-like structures and *r* is the number of residual blocks. $f_{i,j}\in R^{1\times l}$ indicates the *j*-th feature vector generated through ResU Block. *l* represents the length of each feature vector, set to 128. The total feature vectors are stacked into feature tokens $F_{i}\in R^{n\times l}$ in chronological order.

(2) $$F_{i}=\left[f_{i,1},f_{i,2},\ldots ,f_{i,n}\right]$$

#### 2.2.2. Context-Sensitive Interaction

The CIM is utilized to facilitate the interaction of the contextual features from different cycles and model temporal dependencies for SBP fluctuation patterns. Transformer and patch techniques have recently been widely applied and shown to perform well in the field of time series forecasting [37,38]. The CIM exploits the superiority of the Transformer based on multi-head self-attention in global interactions and modeling the contextual dependence [43]. Therefore, the relationships between each cycle’s feature vector and those of all other cycles can be explored. As the temporal distance between different cardiac cycles increases, the dependencies between PPG and BP may vary widely depending on the environment or activity. However, the self-attention mechanism used in the original Transformer does not explicitly emphasize the distance relationships between tokens and relies on positional encoding to introduce temporal information. In contrast to absolute positional encoding (APE), RPE can encode the relative temporal distance between each pair of tokens to enhance the model’s ability for local patterns [44]. Thus, the CIM cascades several Trans-RPE layers.

The output of IFEM, denoted as $F_{i}=\left[f_{i,1},f_{i,2},\ldots ,f_{i,n}\right]$, serves as the input of the CIM as shown in Figure 2. The *t*-th Trans-RPE layer can computed as follows:

(3) $${\hat{F}}_{i}^{t}=f_{MSA}\left(f_{LN}\left(F_{i}^{t-1}\right)\right)+F_{i}^{t-1}$$

(4) $$F_{i}^{t}=f_{MLP}\left(f_{LN}\left({\hat{F}}_{i}^{t}\right)\right)+{\hat{F}}_{i}^{t}$$

where $f_{MSA}$, $f_{MLP}$, and $f_{LN}$ are the multi-head self-attention mechanism, the multi-layer perceptron, and the layer normalization, respectively. ${\hat{F}}_{i}^{t}\in R^{n\times l}$ and $F_{i}^{t}\in R^{n\times l}$ denote the output of the MSA and the MLP in the *t*-th Trans-RPE layer. For t=1, which denotes the input to the first Trans-RPE layer, we have $F_{i}^{t-1}$ = $F_{i}$.

We used sine and cosine functions to encode position information. The PE function is as follows:

(5) $${\mathrm{PE}}_{2x}\left(j\right)=\sin\left(j/10000^{2x/D_{vector}}\right)$$

(6) $${\mathrm{PE}}_{2x+1}\left(j\right)=\cos\left(j/10000^{2x/D_{vector}}\right)$$

where *j* refers to the position index and temporal order of the feature vector $f_{i,j}$ in $F_{i}$. $D_{vector}$ represents the dimension of each feature vector, which is equal to *l*. *x* denotes the feature dimension index. Then, the RPE, which indicates the position of the feature vector of the *a*-th cycle relative to the feature vector of *b*-th, can be defined as follows:

(7) $$p_{a,b}=\mathrm{sign}\left(a-b\right)\mathrm{PE}(|a-b|)$$

where sign denotes the sign function, introducing directionality to the RPE. In particular, RPE incorporates positional differences (relative position) into attention score calculations. Thus, an attention mechanism with positional bias can be formulated as follows:

(8) $$Attention=\mathrm{Softmax}\left(\frac{QK^{T}}{\sqrt{d_{k}}}+W_{p}^{T}p\right)V$$

where $W_{p}^{T}p\in R^{n\times n}$ is the positional bias introduced in the attention score. $W_{p}$ is learnable weights.

Finally, the output projection layer consists of linear layers and activation functions helping translate the high-level feature representations into complex target mappings for more accurate prediction. The projection serves as the final critical step in exploring the relationships between PPG cycles and target SBP trends. Specifically, each PPG cycle has already modeled the interactions through intra-cycle and inter-cycle modules and obtained the corresponding high-level features, which can be mapped to the SBP value of the respective cycle by introducing the Projection. Further, a set of feature vectors $F_{i}\in R^{n\times l}$ obtained from all the consecutive PPG cycles is fed into the projection to generate the SBP sequence $S_{i}\in R^{1\times n}$, as described by the following equation:

(9) $$S_{i}=f_{Projection}\left(F_{i}\right)$$

**Algorithm 1** The architecture of our proposed model**Input:** mini-batch size *B*, training dataset $D={\left\{\left(C_{i},S_{i}\right)\right\}}_{i=1}^{N}$, where $C_{i}\in R^{n\times l_{0}}$ denotes contextual cycles containing *n* cycle slices of length $l_{0}$, and $S_{i}\in R^{n}$ denotes the sequence containing *n* SBP values**Output**: the optimal parameters of model**Initialization**: ResU block, Trans-RPE layers, number of Trans-RPE layers *L*1:**for** $epoch=1,2,\ldots ,N_{epoches}$ **do**2:    (C,S)← sampled mini-batch from *D*3:    **for** i=1,2,…,B **do**4:        $C_{i}\leftarrow \left[c_{i,1},c_{i,2},\ldots ,c_{i,n}\right]$5:        **// Stage 1. Intra-cycle Feature Extraction**6:        **for** j=1,2,…,n **do**7:           // Apply ResU block to the cycle slice $c_{i,j}$ of the contextual cycles $C_{i}$8:           $f_{i,j}\leftarrow \mathrm{ResU}\left(c_{i,j}\right)$9:        **end for**10:        // Concatenates the feature vectors into the feature tokens $F_{i}$11:        $F_{i}\leftarrow \left[f_{i,1},f_{i,2},\ldots ,f_{i,n}\right]$12:        **// Stage 2. Context-sensitive Interaction**13:        $Y\leftarrow F_{i}$14:        **for** t=1,…,L **do**15:           // Apply Trans-RPE to the feature tokens $F_{i}$16:           Y←Trans-RPE(Y)17:        **end for**18:        ${\tilde{S}}_{i}\leftarrow \mathrm{Projection}\left(Y\right)$19:    **end for**20:    $L\left(\theta \right)=\left|\right|\left(S-\tilde{S}\right){\left|\right|}_{1}$21:    Update $\theta^{*}\leftarrow \theta -\alpha \cdot \nabla L\left(\theta \right)$22:**end for**

### 2.3. Experimental Settings

In this architecture, we set the depth of U-like structures *d* and the number of residual blocks *r* to 2 and 8, respectively. In addition, the number of multiple heads in the self-attention and Trans-RPE layers are set to 8 and 3, respectively. Unlike cross-validation, where the dataset is divided into several subsets that are randomly assigned to training and testing folds, our approach explicitly considers the chronological order of the dataset. For each subject, the model is trained on historical data and subsequently tested on future data. This setup aligns with the real-world application scenarios and allows for evaluating the model’s performance in capturing and utilizing temporal dependencies. All the experiments were implemented on a personal computer equipped with a GPU and PyTorch 1.13.1 deep learning framework with Python 3.7. During training, we utilized an Adam optimizer with an initial learning rate of 0.0001, where the batch size is 16 and the epoch number is 500. We employed the MAE as the loss function and conducted an overall evaluation using the following metrics.

### 2.4. Performance Evaluation Metrics

Unlike previous methods, which output a single data point, we estimate SBP trends as a sequence prediction task, where the output is an SBP sequence with multiple SBP values, so we apply the error measurement to the entire sequence using multiple evaluation metrics.

First, MAE can be applied to evaluate the accuracy of the ground truth (GT) and estimated SBP sequences, which is equal to the MAE applied to beat-to-beat SBP values, defined as follows:

(10) $$\mathrm{MAE}=\frac{1}{m}\cdot \frac{1}{n}\sum_{i=1}^{m}\sum_{j=1}^{n}\left|x_{i,j}-y_{i,j}\right|$$

where $x_{i,j}$ is the *j*-th value of the *i*-th GT sequence $X_{i}$, $y_{i,j}$ is the *j*-th value of the *i*-th estimated sequence $Y_{i}$, *n* is the number of SBP values in a SBP sequence, and *m* is the total number of test sequences.

In particular, we assess the correlation between the GT and estimated SBP trends using the Pearson correlation coefficient (R). $R_{\mathrm{seq}}$ applied to the entire sequence is defined as follows:

(11) $$R_{seq}=\frac{1}{m}\sum_{i=1}^{m}\left(\frac{\sum_{i=1}^{n}\left(x_{i,j}-\bar{X_{i}}\right)\left(y_{i,j}-\bar{Y_{i}}\right)}{\sqrt{\sum_{j=1}^{n}{\left(x_{i,j}-\bar{X_{i}}\right)}^{2}}\sqrt{\sum_{j=1}^{n}{\left(y_{i,j}-\bar{Y_{i}}\right)}^{2}}}\right)$$

where $\bar{X_{i}}$ and $\bar{Y_{i}}$ are the means of the *i*-th GT and estimated sequence, respectively.

Independent of average BP, the average real variability (ARV) is defined as the average of the absolute differences between all consecutive BP measurements, which is used to indicate the degree of variability in BP over a period of time [11]. Given the calculation of the first-order difference [45], the differences $D_{i}$ can be derived from the GT sequence $X_{i}$ and its immediately preceding historical time step as follows:

(12) $$D_{i}=\left\{\left(x_{i,2}-x_{i,1}\right),\ldots ,\left(x_{i,n}-x_{i,n-1}\right)\right\}$$

$D_{i}$ can represent the detailed SBP trends, highlighting the direction and magnitude of beat-to-beat SBP changes. Specifically, the magnitude of $\left(x_{i,j}-x_{i,j-1}\right)$ quantifies the increase or decrease in SBP values between two consecutive cycles, while the sign of $\left(x_{i,j}-x_{i,j-1}\right)$ indicates the upward or downward trend between two adjacent cycles. We therefore utilize it to evaluate the performance in SBP trends at a fine-grained level by comparing the direction and magnitude of SBP changes between every two adjacent cycles. For the ground truth and estimated SBP sequences, the variability error (VE) based on ARV is defined as follows:

(13) $$VE=\frac{1}{m}\cdot \frac{1}{n-1}\sum_{i=1}^{m}\sum_{j=2}^{n}\left|\left(x_{i,j}-x_{i,j-1}\right)-\left(y_{i,j}-y_{i,j-1}\right)\right|$$

For SBP trend applications, we detect potential anomalies in several tens of seconds at a coarse-grained level. As a complete surge may exist in several consecutive SBP sequences, and because of the complexity of the detection criteria for the BP surge [46], here, we simplify it to a binary classification of relatively steady-state or abrupt excessive fluctuations to determine whether the overall change in the SBP trends (*ΔSBP*) exceeds a given threshold (here, 10 mmHg) throughout the duration of an SBP sequence.

We adopt precision (Pre), recall (Rec), and F1-score (F1) as per-class metrics to evaluate the classification performance. These metrics are defined as follows:

(14) $$\mathrm{ACC}=\frac{TP+TN}{TP+FP+TN+FN}$$

(15) $$F1=\frac{2\times \mathrm{Precision}\times \mathrm{Recall}}{\mathrm{Precision}+\mathrm{Recall}}$$

where Precision is $\frac{TP}{TP+FP}$, Recall is $\frac{TP}{TP+FN}$, *TP* is true positive, *TN* is true negative, *FP* is false positive, and *FN* is false negative.

**Figure 3:**
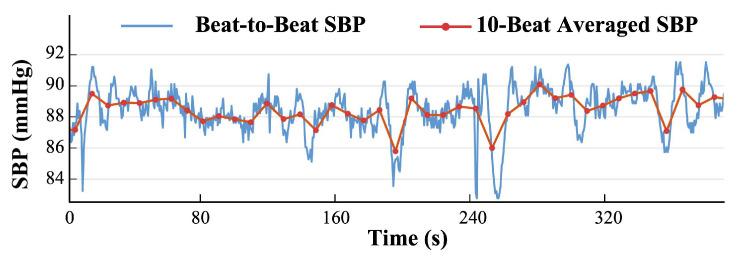
Visualization of beat-to-beat SBP and 10-beat-averaged SBP.

In addition, we evaluate the performance of SBP value prediction; i.e., we evaluate individual data points. Given that most previous works derive their predictions of BP from the average of several consecutive beats, we also assess the accuracy of the beat-averaged SBP for each 10 cycles using several metrics, including MAE, the mean error (ME), and the standard deviation (SD), as shown in Figure 3.

## 3. Results

In this section, we quantitatively and qualitatively analyze the performance of our model in SBP trend estimation and further discuss the performance for the prediction of beat-to-beat and beat-averaged SBP values, as well as the performance comparison with existing methods. The results of the evaluations are reported as follows: (1) SBP trend estimation; (2) SBP value prediction; (3) Comparison with existing methods.

### 3.1. SBP Trend Estimation

Here, we focus on evaluating our performance in SBP trend estimation. Given the advantages of bi-directional long short-term memory and the Transformer with APE (Trans) in modeling temporal dependencies, we consider ResU-LSTM and ResU-Trans (three-layer Trans) as baseline models.

#### 3.1.1. Quantitative Evaluation

We quantitatively evaluate the GT and estimated SBP sequences for very-short-term trends using multiple evaluation metrics. Unlike correlations among all data points, $R_{\mathrm{seq}}$ measures the correlation between two sequences, with values closer to 1 indicating a higher correlation. A lower MAE implies better accuracy of the estimated SBP sequences as it measures the absolute differences between the predicted and actual values at each time step of sequences. Independent of SBP levels, the VE quantifies the errors in the direction and magnitude of SBP changes between every two adjacent cycles of the two sequences. As the VE decreases, the fluctuation patterns and detailed trends of the two sequences align more closely. Table 1 shows the evaluation results of our proposed model. To explore the impact of the number of Trans-RPE *L* on the performance, we conduct comparisons with 1, 2, and 3 layers. Overall, the errors decreased as the number of Trans-RPE *L* was cascaded to 3, with the best MAE, $R_{\mathrm{seq}}$, and VE achieving 3.186, 0.743, and 1.199, respectively. Thus, we deepened the depth of our model with three Trans-RPE layers. The results show a strong correlation and high agreement between the GT and estimated SBP sequences, which suggests the potential ability of our proposed model to track SBP trends.

Table 1 summarizes the performance comparison of baseline models. Specifically, the evaluation of ResU-LSTM reveals a relatively low MAE but higher VE and lower $R_{\mathrm{seq}}$ values, implying that it struggles with capturing the detailed rise/fall relationship of beat-to-beat SBP trends. ResU-Trans performs much better in VE and $R_{\mathrm{seq}}$ relative to ResU-LSTM, showing the advantages of the Transformer in modeling contextual dependencies. Overall, the results illustrate that our proposed model outperforms other baseline models in all metrics.

We implemented a single-beat SBP estimation with one PPG cardiac cycle using solely ResU and the projection layer. MAE, VE, ME, and SD achieve 3.613, 3.661, −0.304, and 6.448, respectively. Notably, although the MAE, ME, and SD achieved satisfactory results, the VE decreased to its lowest point. This emphasizes that the ResU structure is essential for ensuring accuracy, while the patch-based contextual input structure and models with advantages in time series modeling are crucial for effectively exploring BP fluctuation patterns. We further conducted an ablation study on the IFEM of our model and the ResU-Trans, resulting in a decline in performance. Notably, the SD of the remaining CIMs, Trans-RPE and Trans, were 7.222 and 8.358, respectively. This underscores that ResU blocks can effectively capture time-varying features within the cardiac cycle and enhance semantic representations, which are essential for this task. In addition, by comparing our model with ResU-Trans, as well as Trans-RPE with Trans, we observe that models employing RPE consistently outperform those using APE, particularly in terms of VE. It highlights the effectiveness of considering the relative temporal distance and contextual relationships between cardiovascular dynamics in different cycles to explore complex SBP fluctuation patterns. These results demonstrate the superiority and robustness of our model in tracking detailed upward and downward beat-to-beat SBP changes.

**Figure 4:**
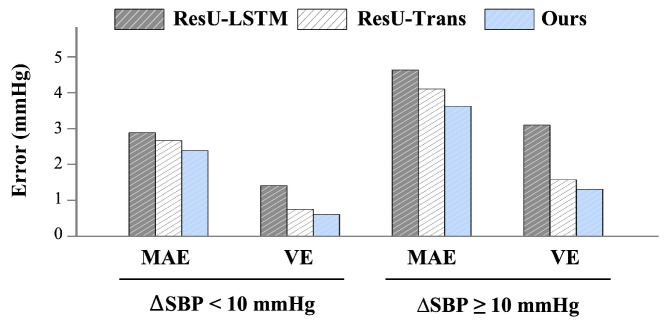
Errors at different states of SBP fluctuations.

To more rationally evaluate SBP values prediction performance at different degrees of SBP changes, we further analyze the error metrics in two states throughout SBP sequences: relatively steady-state (ΔSBP less than 10 mmHg) and abrupt substantial fluctuations (ΔSBP greater than 10 mmHg), as shown in Figure 4. The results clearly show that overall errors in abnormal excessive fluctuations are greater than in relatively steady states, which confirms the heightened difficulty in unsteady states. Regardless of the steady states, the MAE error for each model is slightly different, but VE is notably smaller in ours and ResU-Trans compared to ResU-LSTM. This recognizes the significant contribution of the Transformer and contextual cycles to robust SBP trend estimation. Overall, our model consistently exhibits lower error rates and more robust performance compared to other baseline models.

**Figure 5:**
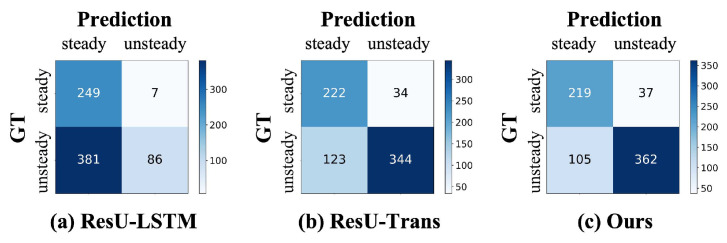
The confusion matrixes of our model and other baseline models in detecting potential anomalies. GT represents the ground truth label of SBP variation states.

In the classification application for detecting abnormalities of extreme SBP changes, a total of 723 SBP sequences were classified as relatively steady states or sharp excessive changes, abbreviated as ‘steady’ and ‘unsteady’. Our classes exhibit a slight imbalance, with the number of instances in the two classes unevenly distributed at 256 and 467 for ‘steady’ and ‘unsteady’, respectively. Figure 5 shows the confusion matrix comparisons with other baseline models. In each confusion matrix, the diagonal entries represent the number of correctly classified instances for each class. The classification results of ResU-LSTM model showed an accuracy of 46.33% and an F1-score of 30.71%. It indicated that the SBP predictions of ResU-LSTM for both ‘steady’ and ‘unsteady’ states are concentrated within a narrow range of 10 mmHg to ensure high stability and precision. Notably, ResU-Trans demonstrates a significant improvement, achieving an overall accuracy of 78.28% and an F1-score of 81.42%. The results indicate that the Transformer effectively learns global information and more flexibly captures contextual dependencies for SBP fluctuations compared to LSTM. Moreover, our proposed method achieves the highest classification performance, with an accuracy of 80.36% and an F1-score of 83.60%. This highlights the potential of our proposed method for detecting abnormalities of SBP changes.

#### 3.1.2. Qualitative Evaluation

**Figure 6:**
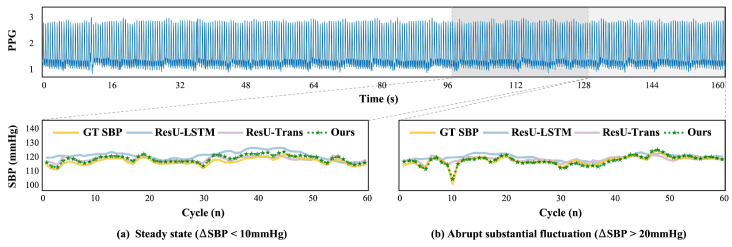
Visualization of the GT and estimated SBP sequences in different states from ours, ResU-LSTM and ResU-Trans. Each subplot below presents the GT (yellow line) and estimated SBP sequences corresponding to the gray box in the figure above (PPG signals). (**a**) Relatively steady state. (**b**) Abrupt excessive fluctuation.

To provide a more intuitive demonstration of the quality of the estimated SBP trends, we present representative observations at different degrees of fluctuations. Figure 6 shows the GT (yellow line) and estimated SBP sequences. Evidently, our estimated SBP sequences (green lines with stars) closely match the yellow lines, even for the abnormal sharp changes. This indicates that our model can stably capture detailed rises and falls, closely approximating the overall range of short-term SBP changes, with the GT and estimated sequences consistently showing similar magnitudes and directions of SBP fluctuations. For relatively steady states, shown in Figure 6a, both ResU-LSTM (blue lines) and ResU-Trans (pink lines) perform well. However, ResU-LSTM fails to track the sharp changes, resulting in the generally smooth trend shown in Figure 6b. Nonetheless, ResU-Trans is somewhat better at capturing abrupt changes than ResU-LSTM. Overall, the qualitative evaluation provides a more intuitive and clear reflection of the error evaluation results above.

### 3.2. SBP Value Prediction

The evaluation results above also partially reflect the performance for the prediction of beat-to-beat SBP values. In addition to beat-to-beat SBP values, recent studies predict beat-averaged SBP values within specific periods (e.g., 5 s/8 s/10 s segments). Consequently, we extend our analysis to assess on the prediction performance for beat-averaged SBP values. Table 2, Table 3, Table 4 show the evaluation results for SBP value prediction. Interestingly, the overall error metrics of our model and other baseline models decrease in the prediction of beat-averaged SBP values compared to beat-to-beat SBP values. This suggests that beat-to-beat SBP exhibits greater fluctuations and complex details, challenging the accurate numerical prediction.

**Figure 7:**
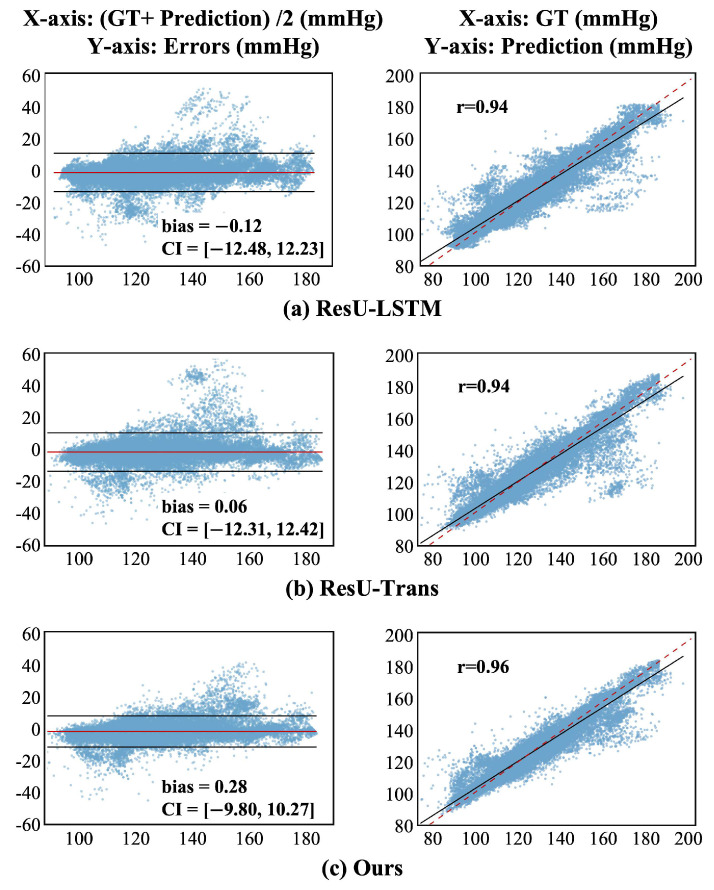
Bland–Altman plot and regression plot of ours and other baseline models.

The Bland–Altman plots and regression plots are given in Figure 7. According to the Bland–Altman recommendation, two methods are considered consistent if 95% of the samples fall within the confidence intervals (CI). The statistics show that most of our SBP predictions and other baseline models fall within the CI out of 43,380 samples. The regression plots, as shown in Figure 7, give an indication of the degree of linear correlation between the GT and prediction, and the Pearson correlation coefficients of our and other baseline models are above 0.94, indicating a high degree of correlation between the predicted and GT SBP values. These results indicate excellent performance across all models in beat-to-beat SBP value prediction. It can be observed in the regression plots that there are some incorrectly predicted results towards the central region in the SBP distribution. This is due to the imbalanced phenomenon caused by the skewed distribution of SBP in the training set, which is a significant challenge in the field of BP monitoring [35]. Compared to other baseline models, our model demonstrates better adaptability to the imbalanced datasets, effectively mitigating the predictive bias to a certain extent.

Moreover, we evaluated our performance in beat-to-beat SBP value prediction using the standards of the Association for the Advancement of Medical Instrumentation (AAMI) and the British Hypertension Society (BHS). According to the AAMI standard, a minimum of 85 subjects is required, and the method can be considered validated if the ME and SD are within the range of 5 mmHg and 8 mmHg, respectively. The BHS standard assigns a performance grade for BP monitoring methods as grade A, B, or C based on the percentage of cumulative errors under the specific threshold (5, 10, and 15 mmHg). Table 2 summarizes a comparison between our results and the criteria required by the AAMI standard. Table 3 reports the performance of our method based on the BHS standard. Our proposed method was evaluated on 238 subjects with an ME of −0.238 mmHg and a SD of 5.120 mmHg, meeting the requirements of the AAMI standard. Also, Table 3 reveals that our method achieves grade A in prediction according to the BHS standard. These results further validate the effectiveness of our method in predicting the beat-to-beat SBP value.

### 3.3. Comparisons

We include recent excellent works and summarize the evaluation results in Table 4 using the MAE, ME, and SD metrics for the two different predictive outputs: beat-to-beat and beat-averaged SBP values. The results of all the compared methods are derived from the original publications, with ‘-’ used to indicate unreported error metrics. The highest score in each comparison metric is highlighted in bold. These existing studies generally adopt single-step prediction, taking the overall signal within a certain period as an input. Since the length of segments varies across different works, we also set 10-beat-averaged SBP values for discussion. Machine learning methods demonstrated promising performance in BP value estimation tasks and offer advantages in terms of low computational resource requirements. However, they often rely on handcrafted features and struggle to consider richer contextual information. Thus, they exhibit limitations in the capacity to explore longer-term temporal dependencies and lead to instability in their performance, particularly during significant BP fluctuations. It can be noted that CNN is prevalently utilized to extract features. The studies applying Residual U-Net, presented in the Table 4, demonstrated the superiority in BP-related feature extraction and multi-level feature fusion of PPG signals. Also, most of these existing studies have used LSTM and GRU for temporal dependence modeling. However, this method is relatively inflexible for complex temporal dependences and the correlation between any two time points. Our proposed method employs ResUnet to extract features and enhance the representation of semantics throughout a complete cycle. Additionally, we incorporate relative positional bias in the Transformer, which increases the flexibility of interaction and improves the ability to adapt to complex dynamics. Further, our proposed model, combined with patch-based input named contextual cycles, shows a better overall performance for SBP value prediction compared to the existing methods.

## 4. Discussion

In this paper, we proposed a novel method for beat-to-beat SBP trend estimation using a patch-based input named contextual cycles. Our method achieves robust trend estimation and effective detection of abnormal fluctuations, not only for accurate SBP values. Sincerely, our goal is to unlock the potential applications of deep learning methods in timely anomaly detection and personalized health monitoring, using wearable devices to continuously acquire PPG data. Significant variations can serve as warning signs to help reduce CVD risks, which is especially crucial for heterogeneous patients with frequent and dramatic fluctuations in SBP. Simultaneously, maintaining BP stability and decreasing the frequency of dramatic changes helps healthy individuals decrease the risk of CVD and provides insights for personalized healthcare.

**Figure 8:**
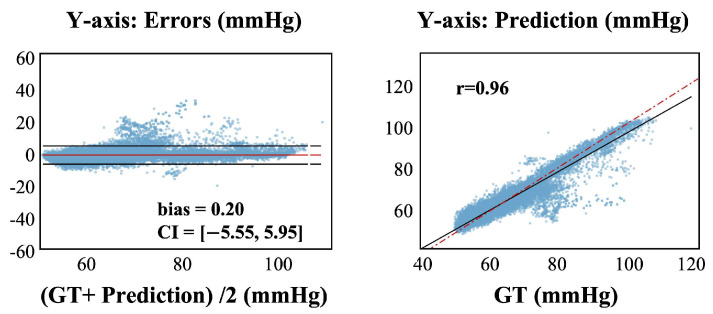
Bland–Altman plot and regression plot of ours in predicting DBP.

In addition, our model demonstrates feasibility in predicting DBP, achieving satisfactory results with MAE, VE, ME, and SD of 1.739, 0.708, −0.203, and 2.934 mmHg, respectively. Figure 8 shows the Bland–Altman plot and regression plot. The results meet the AAMI standard and the BHS criteria for grade A. These results outperform the model’s performance for SBP. The distribution range of DBP is generally narrower than that of SBP, with smaller fluctuations and variability, making the related tasks of SBP inherently complex and clinically challenging.

It is important to provide a summary of this work’s limitations and future directions. While our work demonstrates promising results in estimating SBP trends and detecting abnormal fluctuations, representing a valuable step towards the assessment of beat-to-beat BPV, it is crucial to acknowledge the limitations for a comprehensive BPV evaluation. Indeed, comprehensive BPV assessment is a complex and challenging task, necessitating the consideration of a wider array of physiological signals and analytical approaches. Beyond SBP and DBP, a thorough evaluation may require the integration of other relevant physiological parameters (such as heart rate and respiratory activity) and demands the application of standard relevant metrics, including, but not limited to, the SD and ARV. For example, the accurate identification of SBP surges often necessitates expert-annotated labels based on strict definitions (e.g., magnitude exceeding 20 mmHg, exhibiting a hill-like waveform). In contrast, our approach provides a relatively coarse-grained detection of fluctuations within predefined time windows. Future research can explore novel BP-related methods that capture complex multivariate interactions for comprehensive BPV assessment. Given the strong correlation between SBP and DBP, it is promising to explore their interrelationship to develop a unified model. Furthermore, the absence of clinical validation is a limitation of our study. Nevertheless, we made progress towards practical application by using a dataset rich in BP dynamics, incorporating PPG contextual cycles and a hybrid architecture for waveform nuances and temporal dependencies, and focusing on detailed beat-by-beat trends. As is essential for clinical translation and practical application of continuous BP monitoring, future work can explore strategies like transfer learning or fine-tuning techniques for diverse patient populations, integrate signal quality assessment, and develop noise-robust models.

## 5. Conclusions

In this work, we proposed a hybrid architecture based on contextual cycles for beat-to-beat SBP trend estimation. The fine-grained features of intra-cycle blood flow information were extracted from each cycle of contextual cycles through parallel Residual U-like Blocks in IFEM. A Transformer with RPE was leveraged to model SBP fluctuation patterns by interacting with contextual information from inter-cycle features. We evaluated our proposed method in the performance of SBP trend estimation and SBP value prediction. The overall performance was enhanced when the contextual cycles were combined with our proposed model, which significantly contributed to temporal modeling for beat-to-beat SBP trends. Specifically, our model can more accurately capture the detailed rises/falls than other baseline models and closely approximates the overall degree of SBP fluctuations in the short term.

## Figures and Tables

**Table 1:** Evaluation results of our model and other baseline models for SBP trend estimation.

Model	Metrics (mmHg)		
	MAE	$R_{\mathrm{seq}}$	VE
Trans	5.578	0.520	1.800
Trans-RPE	4.304	0.671	1.318
ResU-LSTM	4.015	0.356	2.500
ResU-Trans	3.595	0.681	1.510
ResU-Trans-RPE (*L*=1)	3.463	0.683	1.464
ResU-Trans-RPE (*L*=2)	3.348	0.706	1.321
**Ours**	**3.186**	**0.743**	**1.199**

**Table 2:** Evaluation results of our model on the AAMI standard.

	ME	SD	Subject
Error standard	≤5 mmHg	≤8 mmHg	85
Ours	−0.238	5.120	238

**Table 3:** Evaluation results of our model on the BHS standard.

	Cumulative Absolute Error Percentage			Grade
	≤**5 mmHg**	≤**10 mmHg**	≤**15 mmHg**	
Our method	82.88%	94.39%	97.43%	A
Grade A	60%	85%	95%	
Grade B	50%	75%	90%	
Grade C	40%	65%	85%	

**Table 4:** Performance comparison of our method with with existing methods for SBP values prediction via deep learning.

Model	Database (No. of Samples)	Input	Beat-to-Beat SBP			Beat-Averaged SBP		
			MAE	ME	SD	MAE	ME	SD
Regression tree [18]	Queensland(32 subjects)	3 features	-	−1.1	5.7	-	-	-
Xgboost [19]	CPT, PPG-BP, and Queensland (327, 840 beats)	15 features	6.37	2.89	12.02	-	-	-
U-Net [47]	MIMIC-II(949 subjects)	10 s PPG segment	5.16	-	-	-	-	-
CNN-LSTM [48]	MIMIC-II(200 subjects)	256-sample PPG segment(contains one complete cardiac cycle)	-	1.91	5.55	-	-	-
Attention-basedresidual improved U-Net [23]	MIMIC-III(100 subjects)	600 samples (PPG, VPG, and APG)(contains 5 consecutive cycles)	-	-	-	4.75	-	6.72
MLPlstm-BP [24]	MIMIC-II (3000 segments)	10 s ECG and PPG segments	-	-	-	3.52	-	5.10
CNN-GRU [26]	MIMIC-III(1293 segments)	10 s ECG and PPG segments	-	-	-	4.90	**0.12**	7.00
SE-MSResUNet [28]	MIMIC-II(111,097 segments)	10 s PPG segment	-	-	-	3.88	-	6.17
**Ours**	MIMIC-II(238 subjects)	contextual cycles(contains 60 complete cardiac cycles)	**3.186**	**−0.238**	**5.120**	**3.053**	−0.238	**4.723**

## Data Availability

The data presented in this study are available in “Cuffless blood pressure estimation algorithms for continuous health-care monitoring” at https://doi.org/10.1109/TBME.2016.2580904. These data were derived from the following resources available in the public domain: https://archive.ics.uci.edu/dataset/340/cuff+less+blood+pressure+estimation (accessed on 26 August 2024).
